# CT-free quantitative SPECT for automatic evaluation of %thyroid uptake based on deep-learning

**DOI:** 10.1186/s40658-023-00536-9

**Published:** 2023-03-22

**Authors:** Kyounghyoun Kwon, Donghwi Hwang, Dongkyu Oh, Ji Hye Kim, Jihyung Yoo, Jae Sung Lee, Won Woo Lee

**Affiliations:** 1 Department of Health Science and Technology, The Graduate School of Convergence Science and Technology, Seoul National University, Suwon, Republic of Korea; 2 Department of Nuclear Medicine, Seoul National University Bundang Hospital, 82, Gumi-ro 173 Beon-gil, Bundang-gu, Seongnam, Gyeonggi-do 13620 Republic of Korea; 3 Department of Biomedical Sciences, Seoul National University, Seoul, Republic of Korea; 4 Department of Nuclear Medicine, Seoul National University Hospital, Seoul, Republic of Korea; 5 Department of Nuclear Medicine, Seoul National University College of Medicine, Seoul, Republic of Korea; 6 Institute of Radiation Medicine, Medical Research Center, Seoul National University, Seoul, Republic of Korea

**Keywords:** Quantification; Single-photon emission computed tomography; Deep-learning; Attenuation correction; Segmentation

## Abstract

**Purpose:**

Quantitative thyroid single-photon emission computed tomography/computed tomography (SPECT/CT) requires computed tomography (CT)-based attenuation correction and manual thyroid segmentation on CT for %thyroid uptake measurements. Here, we aimed to develop a deep-learning-based CT-free quantitative thyroid SPECT that can generate an attenuation map (μ-map) and automatically segment the thyroid.

**Methods:**

Quantitative thyroid SPECT/CT data (*n* = 650) were retrospectively analyzed. Typical 3D U-Nets were used for the μ-map generation and automatic thyroid segmentation. Primary emission and scattering SPECTs were inputted to generate a μ-map, and the original μ-map from CT was labeled (268 and 30 for training and validation, respectively). The generated μ-map and primary emission SPECT were inputted for the automatic thyroid segmentation, and the manual thyroid segmentation was labeled (280 and 36 for training and validation, respectively). Other thyroid SPECT/CT (*n* = 36) and salivary SPECT/CT (*n* = 29) were employed for verification.

**Results:**

The synthetic μ-map demonstrated a strong correlation (*R*^2^ = 0.972) and minimum error (mean square error = 0.936 × 10^−4^, %normalized mean absolute error = 0.999%) of attenuation coefficients when compared to the ground truth (*n* = 30). Compared to manual segmentation, the automatic thyroid segmentation was excellent with a Dice similarity coefficient of 0.767, minimal thyroid volume difference of − 0.72 mL, and a short 95% Hausdorff distance of 9.416 mm (*n* = 36). Additionally, %thyroid uptake by synthetic μ-map and automatic thyroid segmentation (CT-free SPECT) was similar to that by the original μ-map and manual thyroid segmentation (SPECT/CT) (3.772 ± 5.735% vs. 3.682 ± 5.516%, *p* = 0.1090) (*n* = 36). Furthermore, the synthetic μ-map generation and automatic thyroid segmentation were successfully performed in the salivary SPECT/CT using the deep-learning algorithms trained by thyroid SPECT/CT (*n* = 29).

**Conclusion:**

CT-free quantitative SPECT for automatic evaluation of %thyroid uptake can be realized by deep-learning.

**Supplementary Information:**

The online version contains supplementary material available at 10.1186/s40658-023-00536-9.

## Key points

- Question 1: Can CT-free attenuation correction be realized for SPECT?
- Pertinent findings: The first deep-learning algorithm produced μ-map similar to CT-derived μ-map.
- Implications for patient care: Quantitative SPECT can be performed without CT. Therefore, patients can be protected from redundant radiation exposure of CT.
- Question 2: Can the thyroid be segmented without high-resolution images like CT?
- Pertinent findings: The second deep-learning algorithm successfully generated the thyroid segmentation map using low-resolution images such as the generated μ-map and SPECT.
- Implications for patient care: The thyroid segmentation process was dramatically reduced from 40–60 min to < 1 min, facilitating rapid patient care.
- Question 3: Can quantitative SPECT/CT be possible without CT?
- Pertinent findings: The two deep-learning algorithms deprived the quantitative thyroid SPECT/CT of CT.
- Implications for patient care: Repetitive CT acquisitions may be excluded in multiple SPECT/CT-based nuclear imaging studies, such as dosimetry.

**Supplementary Information:**

The online version contains supplementary material available at 10.1186/s40658-023-00536-9.

## Introduction

Attenuation correction (AC) is important for accurate quantitation of radioactivity during single-photon emission computed tomography (SPECT). Attenuation map (μ-map) from x-ray computed tomography (CT) is now technically mature for the purpose of AC. However, application of CT-based AC (CTAC) is yet to be a clinical routine in SPECT because of lack of proper clinical indication, concern about extra-radiation exposure, and necessity for hybrid SPECT/CT scanner [1]. Recent development of deep-learning may change the concept of CTAC because CT acquisition may be omitted through either μ-map generation from SPECT (indirect approach) [2–5] or creation of attenuation-corrected SPECT (direct approach) [6, 7]. Deep-learning was also useful in organ segmentation [8–10].

The rapid progress of deep-learning enables new clinical applications such as CT-free quantitative SPECT as an alternative to quantitative SPECT/CT. The quantitative SPECT/CT is an emerging nuclear medicine technique that employs the AC, scatter correction (SC), and resolution recovery (RR) and provides truly quantitative imaging voxels in units of radioactivity (i.e., kilo Becquerel or micro Curie) [11]. Organ segmentation is also critical in quantitative SPECT/CT because organ-level radioactivity is an important parameter of disease activity (i.e., %thyroid uptake) [8, 10].

Technetium-99m (Tc-99m) is one of the most widely used radionuclides. Currently, quantitative SPECT/CT, which employs Tc-99m, is being actively studied in various conditions, including bone/articular [12–17], parathyroid [18], kidney [8, 19], and salivary gland diseases [10, 20]. Tc-99m pertechnetate uptake in the thyroid gland has been accurately measured using the same principle of quantitative SPECT/CT [21]. Graves' disease [22], chronic thyroiditis [23], and autonomous functional thyroid nodules [24] were evaluated using quantitative thyroid SPECT/CT. However, several limitations exist in clinical application of thyroid SPECT/CT since the thyroid is one of the most sensitive organs to ionizing radiation. Therefore nuclear imaging technique of Tc-99m thyroid uptake measurement can find a broader clinical use if CT is removed according to the ALARA (as low as reasonably achievable) principle without compromising quantitative ability of thyroid SPECT/CT.

CT has twofold application in quantitative thyroid SPECT/CT as follows: attenuation map (μ-map), crucial for attenuation correction of 140 keV photons to accurately quantify Tc-99m thyroid uptake, and thyroid segmentation, which is necessary for automated thyroid uptake evaluation. Here, we developed convolutional neural networks (CNNs) that can remove CT from thyroid SPECT/CT. Specifically, this study aimed to develop a deep-learning-based CT-free quantitative SPECT for %thyroid uptake measurement.

## Materials and methods

### Dataset

In this study, two datasets of thyroid SPECT/CT cases were used. The first SPECT/CT dataset (*n* = 298) was obtained between February 2016 and April 2020, and SPECT and CT covered the same axial field of view (FOV) of 38 cm from the mid-skull to upper mediastinum with the thyroid in centre. The second SPECT/CT dataset (*n* = 352) was obtained between June 2020 and December 2021, and CT did not cover the full but partial axial FOV (approximately 1/2–2/3) of SPECT to reduce redundant radiation exposure. The demographic characteristics of the two datasets were comparable (Table 1). The clinical diagnosis, which was cause of thyroid SPECT/CT referral, was determined by a nuclear medicine physician (DGO) in consideration of thyroid function tests and medical records.

**Table 1:** Demographics of thyroid SPECT/CT cases

	With full CT (*n* = 298)	With partial CT (*n* = 352)	*P* value
Age [years] (mean ± std^a^)	47.6 ± 15.8	47.6 ± 15.6	0.8169 (using unpaired t test)
Sex (male:female)	91:207	99:253	0.5009 (using the Chi-square test)
*Clinical diagnosis*			
Graves’ disease/hyperthyroidism	174	198	0.9062 (using the Chi-square test)
Painless/subacute thyroiditis	96	123	
SNG^b^/MNG^c^	15	17	
Others	13	14	

^a^Standard deviation

^b^Single nodular goiter

^c^Multi-nodular goiter

The first dataset was used for generation of synthetic μ-map (268 and 30 for training and validation, respectively), whereas the second dataset was employed for automatic thyroid segmentation (280 and 36 for training and 36 validation, respectively). The remaining 36 cases in the second dataset were used for an internal verification test to validate both synthetic μ-map generation and automatic thyroid segmentation. In addition to thyroid SPECT/CT, 29 salivary gland SPECT/CT cases were enrolled from the same hospital. The acquisition protocols for thyroid SPECT/CT and salivary SPECT/CT were similar, except for fasting state (no diet restriction vs. fasting for at least 2 h), Tc-99m pertechnetate activity (185 MBq vs. 555 MBq), and organ at the central FOV (thyroid vs. salivary glands) (Additional file 1). Salivary SPECT/CT cases were used as external verification tests for deep-learning algorithms trained by thyroid SPECT/CT.

The details of the 650 thyroid SPECT/CT cases within individual datasets based on the training and validation groups are shown in Additional file 1: Tables S1 and S2. In addition, details of 29 salivary SPECT/CT cases are presented in Additional file 1: Table S3.

### Study scheme

**Fig. 1:**
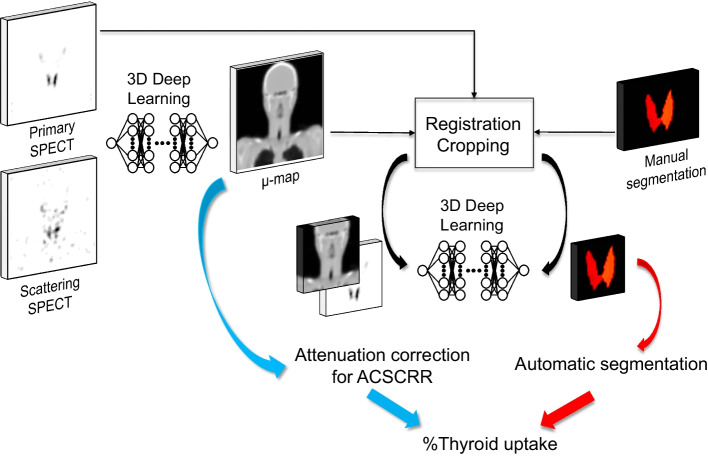
Study scheme

The overall scheme of study is shown in Fig. 1. Two deep-learning algorithms were applied to μ-map generation and automatic thyroid segmentation. The SPECT input of the first deep-learning algorithm for μ-map generation was either only primary emission SPECT (p) or a combined primary emission and scattering SPECTs (ps). The label was original μ-map created with helical CT of SPECT/CT. The generated μ-map was used for attenuation correction (AC) of primary emission SPECT (curved blue arrow). In addition to AC, scatter correction (SC) and resolution recovery (RR) were applied, which resulted in quantitative ACSCRR SPECT (Q.VolumetrixMI, GE). The second deep-learning algorithm was trained for automatic thyroid segmentation using synthetic μ-map input with SPECT support (curved red arrow). The SPECT support was investigated for p, ps, and CT-free quantitative ACSCRR SPECT. The label for automatic thyroid segmentation was the thyroid segmentation map drawn on CT by two human experts (JHK and JHY). Finally, the CT-free quantitative ACSCRR SPECT and automatically segmented thyroid map were combined to calculate the %thyroid uptake (straight blue and red arrows).

### Pre-processing for deep-learning

The thyroid SPECT/CT acquisition protocol and quantification process have already been published in previous studies [21–24] and are described in Additional file 1.

SPECT reconstruction processes were conducted using vendor-provided quantitative software (Q.VolumetrixMI, GE) and the ordered-subsets expectation–maximization (OSEM) iteration algorithm (4 iterations and 10 subsets). The matrix and voxel sizes were 128 × 128 × 128 and 2.95 × 2.95 × 2.95 mm^3^, respectively, for the SPECT images. The primary emission and scattering SPECTs were not corrected for attenuation or scattering but rather for collimator-detector response (i.e., resolution recovery, RR), resulting in NCRR SPECT. In addition, a post-reconstruction Butterworth low-pass filter (order of 10 and cutoff frequency of 0.48 cycles/cm) was used for scattering SPECT to reduce statistical noise.

For a μ-map generation, primary emission and scattering SPECT images were normalized by the maximum of two SPECTs’ summed images. The voxel value was set to 0 in negative value voxels to reduce possible noisy random error during SPECT reconstruction. For automatic thyroid segmentation, synthetic μ-map and SPECT support were cropped from 128 × 128 × 128 to 64 × 64 × 64 to save training time and resources and were subsequently normalized by maximum value to the input range of 0–1. The manual thyroid segmentation map’s initial matrix and voxel sizes were 256 × 256 × slice and 1.47 × 1.47 × 1.47 mm^3^, respectively, which were down-sampled to 128 × 128 × 128 and 2.95 × 2.95 × 2.95 mm^3^ and subsequently cropped to 64 × 64 × 64 for consistency with the synthetic μ-map and SPECT support.

### Network architecture and loss function for μ-map generation

We used a standard 3D U-Net with 64 initial neurons and 4 skip connections. The 3D U-Net learns end-to-end for μ-map generation between SPECTs (primary emission and scattering) and original μ-map. During the contraction path, 3 × 3 × 3 convolution blocks were applied, followed by 2 × 2 × 2 strided max-pooling. Individual 3 × 3 × 3 convolution blocks comprised two times 3 × 3 × 3 convolutions, instance normalization, and rectified linear unit activation. During the extraction path, 3 × 3 × 3 convolution blocks were followed by 2 × 2 × 2 up-convolution. Notably, the last 3 × 3 × 3 convolution block led to 1 × 1 × 1 convolution without an activation function (Additional file 1: Fig. S1).

The loss function for μ-map generation was defined as follows:

$$L\left(G(X), Y\right)={L}_{\mathrm{error}}\left(G\left(X\right), Y\right)+{L}_{\mathrm{GDL}}(G\left(X\right), Y)$$

where *Y* is the target (i.e., original CT-based μ-map) and *G*(*X*) are synthetic μ-maps generated from SPECT input *X*. *L*_error_ is either *L*_1_ loss (i.e., sum of the absolute differences between the target and generated) or *L*_2_ loss (i.e., sum of the squared differences between the target and generated). *L*_GDL_ is gradient difference loss (GDL) term for sharpening the generated μ-maps (Additional file 1).

### Network architecture and loss function for automatic thyroid segmentation

A similar 3D U-Net was used for automatic thyroid segmentation. The 3D U-Net used batch normalization rather than instance normalization and had an additional softmax activation following the last 1 × 1 × 1 convolution (Additional file 1: Fig. S2). The right and left thyroid lobes were individually segmented; thus, the loss function for automatic thyroid segmentation was a categorical cross-entropy (CCE) loss. The CCE loss was defined as follows:

$$\mathrm{CCE}=-\frac{1}{n}\sum_{i=1}^{n}[{y}_{i}\mathrm{log}\left({\widehat{y}}_{i}\right)+\left(1-{y}_{i}\right)\mathrm{log}(1-{\widehat{y}}_{i})]$$

where ${y}_{i}$ is 0 or 1 as the ground truth, ${\widehat{y}}_{i}$ is the probability of prediction, and *n* is the number of classes in the segmentation model. Here, the network generated three independent binary segmentation masks (background, left thyroid lobe, and right thyroid lobe; *n* = 3).

We implemented our networks using TensorFlow [25] and Keras framework [26].

### Training hyperparameters

Both μ-map generation and automatic thyroid segmentation used similar training hyperparameters. The batch size was 8. Furthermore, an adaptive moment estimation optimizer was used. As the learning rate scheduler, the initial learning and exponential decay rates were 0.001 and 0.99, respectively. We also applied data augmentation through flips. For μ-map generation, the SPECT input images were flipped along the *x*, *y*, and *z* axes; while, for automatic thyroid segmentation, the input images (synthetic μ-map and primary emission SPECT) were flipped along the *x* and *z* axes. The intended total number of epochs was 100, and early stopping was applied. The training time was approximately 15 min/epoch and 5 min/epoch for μ-map generation and automatic thyroid segmentation, respectively, with an AMD Ryzen7 5800X CPU and an NVIDIA RTX 3090 GPU.

### Evaluation of outcomes

The attenuation coefficient has units of cm^−1^, and the correlations of attenuation coefficients between the synthetic μ-map and original μ-map were evaluated as *R*^*2*^, mean square error (MSE), and %normalized mean absolute error (%NMAE) (Additional file 1). The manual and automatic thyroid segmentation agreement was analyzed using the Dice similarity coefficient (DSC) (Additional file 1). In addition, the thyroid volume difference (automatic thyroid volume–manual thyroid volume) was calculated, and the 95% Hausdorff distance was used to indicate surface contour difference (Additional file 1). Finally, the %thyroid uptake of Tc-99m pertechnetate, the ultimate parameter of quantitative SPECT, was compared between CT-free thyroid SPECT (attenuation correction by synthetic μ-map and automatic thyroid segmentation) and conventional thyroid SPECT/CT (attenuation correction by original μ-map and manual thyroid segmentation).

### Internal and external verification tests

We recruited 36 thyroid SPECT/CT cases with partial CT that were not applied in the μ-map generation or automatic thyroid segmentation for the internal verification test (Additional file 1: Table S2). Using the first deep-learning algorithm, the ps SPECTs generated μ-map. Subsequently, the generated μ-map and p SPECT produced thyroid segmentation map using the second deep-learning algorithm. Additionally, quantitative ACSCRR SPECT images were reconstructed using synthetic μ-map-based AC, SC, and RR. Then, the %thyroid uptake was calculated by applying the automatic thyroid segmentation map to quantitative ACSCRR SPECT. All processes were performed without CT assistance (i.e., CT-free SPECT) (Fig. 4). On the other hand, with CT assistance, the conventional quantitative ASCSRR SPECT was reconstructed using original μ-map-based AC, SC, and RR (SPECT/CT). Then, the thyroid was segmented by a human expert (JHK) on CT, and the ground truth %thyroid uptake was obtained.

External verification tests were performed on the salivary gland SPECT/CT (*n* = 29) using the same radiotracer (Tc-99m pertechnetate) as in the thyroid SPECT/CT. Here, patients fasted for at least 2 h, the injected radioactivity was three times that of thyroid SPECT/CT, and the salivary glands, instead of the thyroid, were located at the centre of the FOV. Otherwise, the acquisition protocol for the salivary SPECT/CT was the same as that for the thyroid SPECT/CT (Additional file 1). Primary emission and scattering salivary SPECTs were reconstructed using the same reconstruction algorithms as that of the thyroid SPECT (Q.VolumetrixMI, GE) and were used as inputs to generate μ-maps (Additional file 1: Fig. S1). Then, the generated μ-maps were used with the primary emission salivary SPECT as input for automatic thyroid segmentation (Additional file 1: Fig. S2). Next, a human expert (JHK) manually segmented the thyroid from the CT of the salivary gland SPECT/CT. Quantitative ACSCRR SPECT images were reconstructed for CT-free SPECT and SPECT/CT.

### Statistical analysis

Parametric analyses (paired *t*, unpaired *t*, and analysis-of-variance tests) were performed for continuous variables when Shapiro–Wilk test did not reject normal distribution features. Non-parametric Wilcoxon rank-sum test was performed when paired t test was not appropriate owing to rejection of normal distribution assumption. Furthermore, categorical variables were compared using the Chi-square test. Statistical significance was set at *p* < 0.05. All analyses were performed using statistical software (MedCalc, version 20.110).

## Results

### μ-map generation

**Fig. 2:**
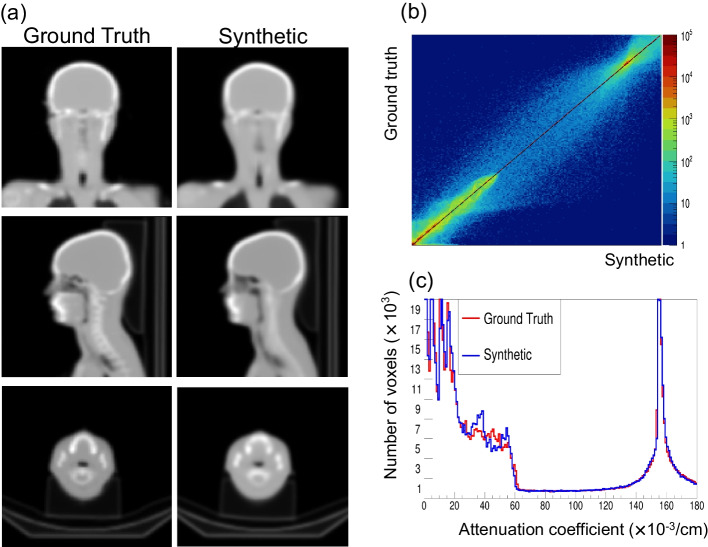
The generation of attenuation map (μ-map) by deep-learning. **a** The ground truth (original μ-map from CT) and synthetic μ-map. **b** Correlation plot and **c** Histogram of attenuation coefficients

Among 298 thyroid SPECT/CT cases with full CT coverage, 268 and 30 were used for training and validation, respectively. There were no age, sex, or clinical diagnosis differences between the training and validation groups (Additional file 1: Table S1). We tested different loss functions (*L*_1_ + *L*_GDL_ vs. *L*_2_ + *L*_GDL_) and SPECT inputs (p vs. ps). Consequently, the 3D U-Net produced almost identical μ-maps as the original. Furthermore, applying the *L*_1_ + *L*_GDL_ loss function and primary emission and scattering SPECTs (ps SPECTs) input yielded the highest *R*^2^ and lowest MSE/%NMAE (Table 2), although the predicted μ-maps by different combinations of losses (*L*_1_ + ps, *L*_2_ + p, *L*_2_ + ps, *L*_2_ + p) did not demonstrate significant visualization results (Additional file 1: Figs. S3 and S4). Therefore, the 3D U-Net trained with the *L*_1_ + *L*_GDL_ loss function and ps SPECT inputs was subsequently used to generate the μ-map. Figure 2 shows the strong correlation between the ground truth (original μ-map) and the synthetic μ-map in one of the 30 validation cases.

**Table 2:** The correlations of attenuation coefficients between original attenuation map (μ-map) (ground truth) and synthetic μ-map (*n* = 30)

Metric	*L*_1_^c^ + *L*_GDL_^d^ with ps^h^			*L*_1_ + *L*_GDL_ with p^i^			*L*_2_^e^ + *L*_GDL_ with ps			*L*_2_ + *L*_GDL_ loss with p		
	*R*^2^	MSE^f^ (× 10^−4^)	%NMAE^g^	*R*^2^	MSE (× 10^−4^)	%NMAE	*R*^2^	MSE (× 10^−4^)	%NMAE	*R*^2^	MSE (× 10^−4^)	%NMAE
Mean	0.972	0.936	0.999	0.972	0.939	1.002	0.971	0.977	1.143	0.971	0.961	1.143
STD^a^	0.012	0.425	0.262	0.012	0.447	0.274	0.012	0.426	0.282	0.012	0.426	0.282
SER^b^	0.002	0.078	0.048	0.002	0.081	0.050	0.002	0.078	0.052	0.002	0.078	0.052

^a^Standard deviation

^b^Standard error

^c^The sum of the absolute differences between the target and generated

^d^Gradient difference loss

^e^The sum of the squared differences between the target and generated

^f^Mean square error

^g^Normalized mean absolute error

^h^Primary emission SPECT + scattering SPECT

^i^Primary emission SPECT

### Automatic thyroid segmentation

Automatic thyroid segmentation was performed on 316 (280 and 36 for network training and validation, respectively) of the 352 thyroid SPECT/CT cases with partial CT coverage. No differences in age, sex, or clinical diagnosis between the training and validation groups were observed, which was similar to that during the μ-map generation (Additional file 1: Table S2). We examined the synthetic μ-map input with SPECT support, which comprised p, ps, and CT-free quantitative ACSCRR SPECT.

**Fig. 3:**
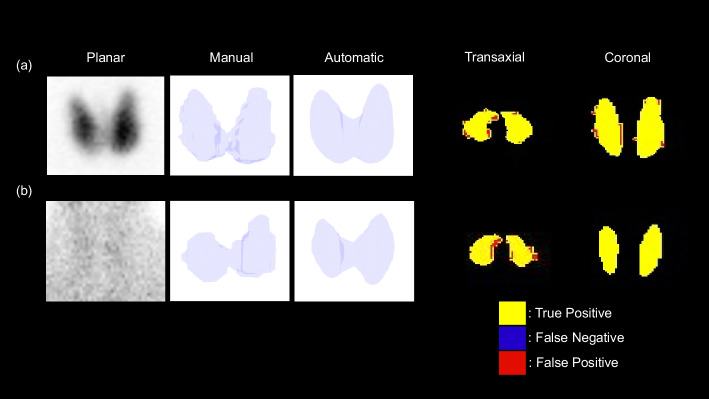
The automatic thyroid segmentation by deep-learning. **a** Patient with Graves’ disease (F/32) with high uptake of Tc-99m pertechnetate. **b** Patient with subacute thyroiditis (F/25) with faint uptake of Tc-99m pertechnetate

**Fig. 4:**
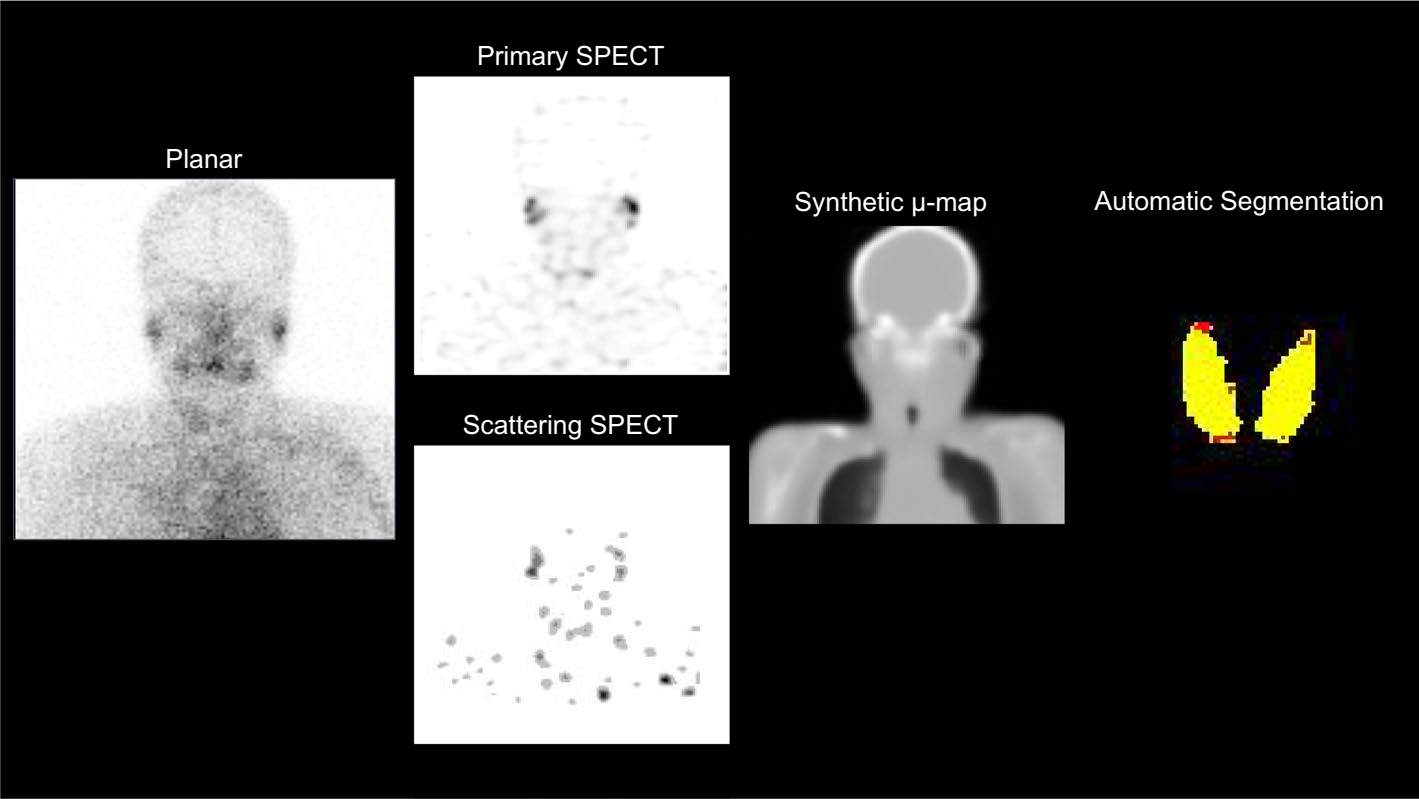
CT-free quantitative thyroid SPECT in a patient with thyroiditis (F/57). A planar scan shows a faint thyroid uptake. Primary emission SPECT and scattering SPECT were used for μ-map generation. Subsequently, the synthetic μ-map and primary emission SPECT were used for automatic thyroid segmentation. Yellow and red indicate true and false positives of the automatic thyroid segmentation, respectively, compared with manual segmentation. The %thyroid uptake by CT-free SPECT was 0.11%, whereas that by conventional SPECT/CT was 0.08%, consistent with the clinical condition of thyroiditis and faint uptake in the planar scan. The reported normal reference range was 0.78 ± 0.5%

The results showed that synthetic μ-map input with p was sufficient for the automatic thyroid segmentation with a large DSC of 0.767, the least absolute thyroid volume difference of − 0.720 mL, and the shortest 95% Hausdorff distance of 9.416 mm (Table 3). Both ps SPECTs and ACSCRR SPECT were inferior to p, particularly for the thyroid volume difference and 95% Hausdorff distance (Table 3). Both the hyperthyroidism and thyroiditis cases readily exhibited successful thyroid segmentation (Fig. 3). Notably, the surface of the segmentation map became smooth through deep-learning. Furthermore, human experts spent approximately 40–60 min per case on manual thyroid segmentation, whereas automatic thyroid segmentation took less than a minute (Fig. 4).

**Table 3:** The automatic thyroid segmentation outcomes according to SPECT input conditions in addition to the attenuation map (μ-map) input (*n* = 36)

Metric	μ-map + p^g^			μ-map + ps^h^			μ-map + ACSCRR^f^			μ-map only		
	DSC^c^	VD^d^ (mL)	95% HD^e^ (mm)	DSC	VD (mL)	95% HD (mm)	DSC	VD (mL)	95% HD (mm)	DSC	VD (mL)	95% HD (mm)
Mean	0.767	− 0.720	9.416	0.768	2.345	10.030	0.769	1.423	10.441	0.727	− 5.239	10.050
STD^a^	0.072	6.661	3.604	0.077	6.952	3.624	0.075	7.363	5.530	0.067	8.067	2.916
SER^b^	0.012	1.110	0.607	0.013	1.159	0.604	0.012	1.227	0.922	0.011	1.344	0.486

^a^Standard deviation

^b^Standard error

^c^Dice similarity coefficient

^d^Volume difference

^e^Hausdorff distance

^f^Quantitative SPECT with attenuation correction, scatter correction, and resolution recovery

^g^Primary emission SPECT

^h^Primary emission SPECT + scattering SPECT

### Internal verification

**Fig. 5:**
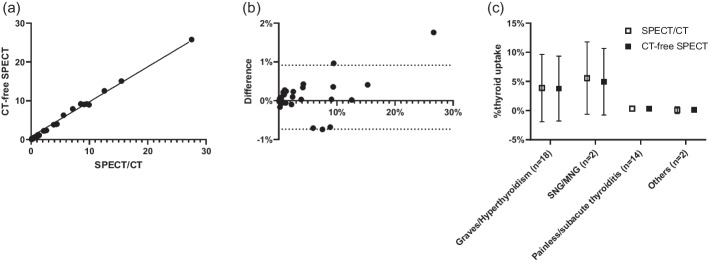
%thyroid uptake between CT-free SPECT and SPECT/CT as an internal verification test (*n* = 36 thyroid SPECT/CT). **a** The correlation is excellent with *r* = 0.9980, *R*^2^ = 0.9959, and *p* < 0.0001. **b** The Bland–Altman plot shows no significant systemic deviation with bias =  − 0.09% point. **c** The %thyroid uptake readily differentiates the thyroid diseases. Data are mean ± standard deviation. The other two cases are drug-induced thyroiditis and lingual thyroid. The error bars for painless/subacute thyroiditis and the others are not obvious because of the limited size compared with the respective symbols. The normal reference range was reported as 0.78 ± 0.5% [21]. SNG, single nodular goiter and NMG, multi-nodular goiter

As shown in Fig. 2, the generated μ-maps were almost identical to those of the ground-truth, and the automatic thyroid segmentation was strongly correlated with the manual thyroid segmentation (Fig. 3, Table 4). Furthermore, the thyroid-specific ACSCRR SPECT counts were strongly correlated with each other (Table 4), and no significant difference was observed in the %thyroid uptake between CT-free SPECT and SPECT/CT (3.772 ± 5.735% vs. 3.682 ± 5.516%, *p* = 0.1090). Moreover, the % thyroid uptakes by both SPECTs were also strongly correlated, rarely biased, and easily differentiated thyroid diseases (Fig. 5).

**Table 4:** The internal verification of CT-free SPECT versus SPECT/CT (*n* = 36)

Metric	Thyroid segmentation			Thyroid-specific SPECT counts		
	DSC^c^	VD^d^ (mL)	95% HD^e^ (mm)	*R*^2^	MSE^f^ (× 10^−4^)	%NMAE^g^
Mean	0.756	1.371	12.063	0.963	0.061	0.002
STD^a^	0.072	8.732	6.445	0.026	0.115	0.001
SER^b^	0.012	1.455	1.074	0.004	0.019	0.000

^a^Standard deviation

^b^Standard error

^c^Dice similarity coefficient

^d^Volume difference

^e^Hausdorff distance

^f^Mean square error

^g^Normalized mean absolute error

### External verification

**Fig. 6:**
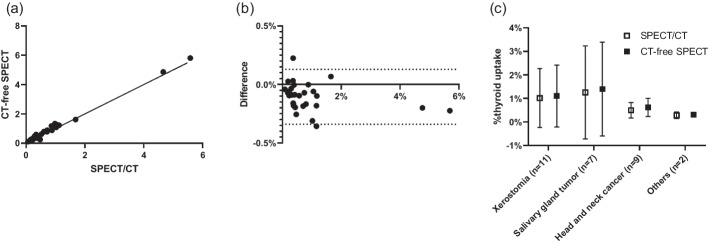
%thyroid uptake between CT-free SPECT and SPECT/CT as an external verification test (*n* = 29 salivary SPECT/CT). **a** The correlation is excellent with *r* = 0.9959, *R*^2^ = 0.9918, and *p* < 0.0001. **b** The Bland–Altman plot shows mild deviation (greater %thyroid uptake by CT-free SPECT) with bias = 0.106% point. **c** %thyroid uptakes between CT-free SPECT and SPECT/CT were similar in various salivary diseases. The normal reference range of %thyroid uptake was reported as 0.78 ± 0.5% [21]

CT-free SPECT was successful in most salivary SPECT/CT cases through deep-learning which was trained using the thyroid SPECT/CT (Table 5 and Additional file 1: Fig. S5). The generated μ-map was identical to that shown in Fig. 2, but automatic thyroid segmentation yielded a larger thyroid volume (26.252 ± 12.023 mL) than manual thyroid segmentation (18.772 ± 8.407 mL) (*p* < 0.0001). Accordingly, the %thyroid uptake on CT-free SPECT (0.939 ± 1.266%) was greater than that on SPECT/CT (0.851 ± 1.223%) (*p* = 0.0035). However, the strong correlation of %thyroid uptake between CT-free SPECT and SPECT/CT was still observed, and in various salivary diseases, the %thyroid uptakes by both SPECTs were highly comparable with only mild deviation (Fig. 6). One patient had concomitant Graves’ disease with high %thyroid uptake (4.862% and 4.662% on CT-free SPECT and SPECT/CT, respectively) (Additional file 1: Fig. S6), and the other 28 were euthyroid patients (0.828 ± 1.055% and 0.726 ± 1.024% by CT-free SPECT and SPECT/CT, respectively) (*p* = 0.0002). CT-free SPECT and SPECT/CT were similar in differentiating between hyperthyroidism and euthyroidism, considering the reported normal range of %thyroid uptake (0.78 ± 0.5%) [21].

**Table 5:** The external verification of CT-free SPECT versus SPECT/CT (*n* = 29 salivary gland SPECT/CT)

Metric	μ-map generation			Thyroid segmentation			Total SPECT counts			Thyroid-specific SPECT counts		
	*R*^2^	MSE^c^ (× 10^−4^)	%NMAE^d^	DSC^e^	VD^f^ (mL)	95% HD^g^ (mm)	R^2^	MSE (× 10^−4^)	%NMAE	R^2^	MSE (× 10^−4^)	%NMAE (× 10^−3^)
Mean	0.942	1.875	1.834	0.657	7.480	13.514	0.983	0.066	0.044	0.992	0.010	0.778
STD^a^	0.020	0.621	0.614	0.088	7.383	7.213	0.010	0.035	0.025	0.019	0.030	0.615
SER^b^	0.004	0.113	0.112	0.016	1.348	1.317	0.002	0.006	0.005	0.003	0.005	0.112

^a^Standard deviation

^b^Standard error

^c^Mean square error

^d^Normalized mean absolute error

^e^Dice similarity coefficient

^f^Volume difference

^g^Hausdorff distance

## Discussion

Radioactive iodine uptake (RAIU) has been used for decades in conventional nuclear medicine practice to quantitatively evaluate thyroid function [27]. Technetium thyroid uptake (TcTU) has been widely investigated as a surrogate for RAIU because it is facile, fast, and inexpensive with lower radiation exposure to patients than the RAIU [28–31]. In contemporary nuclear medicine practice, quantitative thyroid SPECT/CT, which employs AC, SC, and RR, has emerged as the most sophisticated method for measuring %thyroid uptake [21]. Therefore, the %thyroid uptake and the standardized uptake value (SUV) could be examined for functioning thyroid diseases in the quantitative SPECT/CT era [22–24].

However, CT acquisition remains a significant barrier to the widespread clinical application of quantitative thyroid SPECT/CT because of CT-induced radiation exposure. Additionally, the time-consuming manual thyroid segmentation on CT canvas is challenging. In the literature, there are deep-learning-based CT-free AC studies for myocardial perfusion SPECT [2, 5], brain perfusion SPECT [6, 7, 32] and dopamine-transporter brain SPECT [3]. Undoubtedly, AC using CT is essential for quantitative thyroid SPECT/CT, but thyroid-dedicated deep-learning study has not been investigated. Therefore, this study attempted to address the CT-related issues associated with quantitative thyroid SPECT/CT.

We discovered that the deep-learning-derived μ-maps are almost identical to those derived from CT. The *L*_1_ + *L*_GDL_ loss function was superior to *L*_2_ + *L*_GDL_ loss function in terms of network training, which is in line with recent report favouring *L*_1_ loss over *L*_2_ loss regarding CT image reconstruction [33]. Notably, deep-learning enabled the ps SPECTs to generate μ-map, as reported in a myocardial perfusion SPECT/CT study [2]. The previous study demonstrated the accuracy of deep-learning-based AC in the myocardial perfusion SPECT, while we verified the accuracy in the quantitative thyroid SPECT. A similar concept was initially examined using positron emission tomography/computed tomography (PET/CT). Generating μ-map using deep-learning has been reported in fluorodeoxyglucose (FDG) brain PET/CT [34], fluoropropyl carbomethoxy iodophenyl tropane (FP-CIT) brain PET/CT [35], and FDG whole-body PET/CT [36, 37], resulting in CT-free PET. Notably, only primary emission coincidence data were used to generate μ-map rather than scattering coincidence data in those PET studies [34–37]. In PET, the scattering coincidence data cannot be properly estimated without μ-map. Therefore, using scattering information to predict the μ-map is challenging in principle. However, scattering information is relatively easy to obtain in SPECT by simply setting up an additional energy window. Therefore, CT-free SPECT by deep-learning has technical advantages over CT-free PET.

Deep-learning-based organ segmentation on CT has been actively investigated for single [8, 10] or multiple organs [38, 39]. Thyroid segmentation has been a major concern by radio-oncologists seeking to save the thyroid from external radiation therapy for head and neck cancer. CT with or without iodine contrast was generally used as the input for network training [40–42]. It is of note that this study employed synthesized μ-map rather than CT as the input for thyroid segmentation. Low-resolution images, such as μ-map, can be used as input for deep-learning-based automatic organ segmentation. The μ-map alone was clearly insufficient, and SPECT support was required to improve the segmentation results (Table 3), providing insight into the deep-learning-based organ segmentation mechanism. We believe that the μ-map provides a contour of the head and neck for determining the approximate location of the thyroid, while the SPECT signal confirms its presence. Although Tc-99m pertechnetate uptake was low and faint in patients with thyroiditis, it was sufficient evidence of thyroid existence (Figs. 3, 4).

The use of the salivary gland SPECT/CT as external verification test is a characteristic advantage of thyroid SPECT/CT with some theoretical pitfalls. Same radiopharmaceutical (i.e., Tc-99m pertechnetate), same interval from injection to imaging (i.e., 20 min), same time of image acquisition (i.e., 1 min), same SPECT reconstruction, same CT acquisition conditions, and almost equivalent imaging field-of-view (i.e., head and neck area) were the common features between the two SPECT/CTs. The radioactivity for the salivary gland SPECT/CT (555 MBq) was three times that for thyroid SPECT/CT (185 MBq) and this may explain the volume difference of segmented thyroid (Table 5). However, the ultimate quantitative parameter of %thyroid uptake from the CT-free salivary gland SPECT was comparable to that from the CT-free salivary gland SPECT (Fig. 6).

High-resolution images, such as synthetic CT (or pseudo-CT), can be generated as intermediates for thyroid segmentation instead of the μ-map [43, 44]. Then, another μ-map generation process is necessary for the AC. In contrast, fully attenuation-corrected SPECT can be obtained without intermediate μ-map generation, such as direct conversion from non-attenuation-corrected PET to attenuation-corrected PET [45]. In this case, another method for automatic thyroid segmentation is required. In this regard, we expect that using a μ-map as a bridge between two deep-learning networks (one for AC and the other for automatic organ segmentation) would minimize the overall effort required to evaluate %thyroid uptake.

## Conclusion

Sequential application of two deep-learning algorithms (the former for synthetic μ-map generation from SPECT images and the latter for automatic thyroid segmentation from the generated μ-map with primary emission SPECT support) can realize CT-free quantitative SPECT for %thyroid uptake.

## Supplementary Information

**Additional file 1.** Supplemental figures, tables and detailed methods
