# Supplementary material for: CT-free quantitative SPECT for automatic evaluation of %thyroid uptake based on deep-learning
Source: EJNMMI Phys. 2023 Mar 22;10:20. doi: 10.1186/s40658-023-00536-9 (PMC10033819; doi:10.1186/s40658-023-00536-9)
Supplement: Supplementary file 1 — Additional file 1. Supplemental figures, tables and detailed methods [file 40658_2023_536_MOESM1_ESM.docx]

**CT-free Quantitative SPECT for Automatic Evaluation of %thyroid Uptake based on Deep-learning**

**Journal Name: European Journal of Nuclear Medicine and Molecular Imaging**

Kyounghyoun Kwon, MS^1,2^ Donghwi Hwang, MS^3,4^; Dongkyu Oh, MD^2,5^; Ji Hye Kim, RN^2^; Jihyung Yoo, RN^2^: Jae Sung Lee, PhD^3,4,5,6^; Won Woo Lee, MD, PhD^1,2,5,6^

^1^Department of Health Science and Technology, The Graduate School of Convergence Science and Technology, Seoul National University, Republic of Korea;

^2^Department of Nuclear Medicine, Seoul National University Bundang Hospital, Republic of Korea;

^3^Department of Biomedical Sciences, Seoul National University, Seoul, Republic of Korea;

^4^Department of Nuclear Medicine, Seoul National University Hospital, Seoul, Republic of Korea;

^5^Department of Nuclear Medicine, Seoul National University College of Medicine, Seoul, Republic of Korea;

^6^Institute of Radiation Medicine, Medical Research Center, Seoul National University, Seoul, Republic of Korea.

Fo correspondence contact:

Won Woo Lee, Department of Nuclear Medicine, Seoul National University Bundang Hospital, Republic of Korea.. E-mail: wwlee@snu.ac.kr

**Supplemental Material**

**Supplemental Fig 1.** 3D U-Net architecture for the μ-map generation

**Supplemental Fig 2.** 3D U-Net architecture for the automatic thyroid segmentation


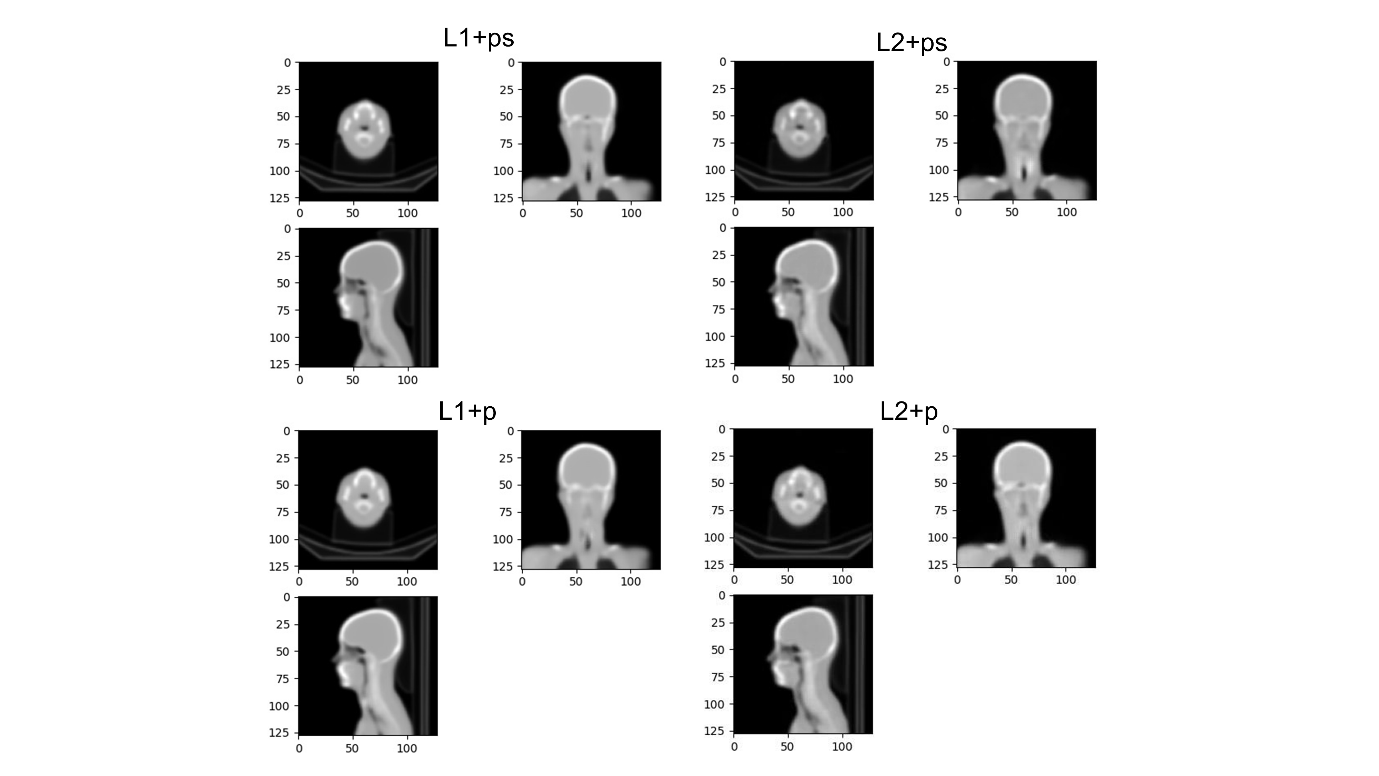


**Supplemental Figure 3**. Synthetic μ-maps


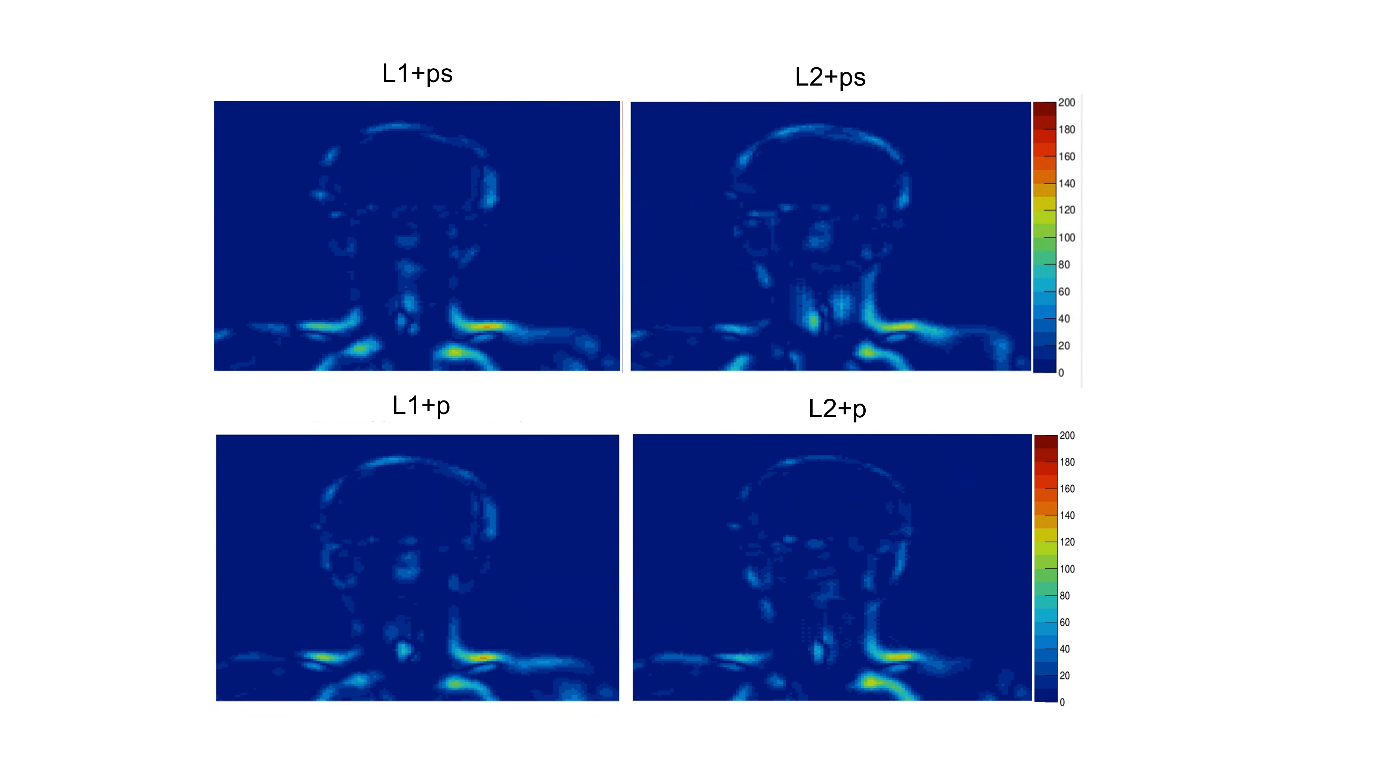


**Supplemental Figure 4**. Absolute error maps between ground truth and synthetic μ-maps

**Supplemental Fig 5.** CT-free salivary SPECT in a euthyroid patient with left hypo-pharyngeal cancer (F/48). (a) Primary emission SPECT and scattering SPECT produced the synthetic μ map. (b) After cropping, the synthetic μ-map and the primary emission SPECT generated the thyroid segmentation map. The %thyroid uptake by deep-learning was normal at 0.886%, similar to 0.718% by conventional SPECT/CT. The normal reference range was 0.78 ± 0.5%.

**Supplemental Fig 6.** CT-free salivary SPECT in a patient with dry mouth and concomitant Graves’ disease (F/56). (a) Primary emission SPECT and scattering SPECT generated the synthetic μ-map. (b) The synthetic μ-map and the primary emission SPECT produced the thyroid segmentation map after cropping. The %thyroid uptake by deep-learning was elevated as 4.862%, which corresponds to that by conventional SPECT/CT (4.662%). The normal reference range was 0.78 ± 0.5%.

Supplementary Table 1. Detailed demographics of thyroid SPECT/CT cases with full CT for attenuation map generation (n = 298)

|  | | For training (n = 268) | For validation  (n = 30) | *P*-value |
| --- | --- | --- | --- | --- |
| Age [years] (mean±std^a^) | | 47.5 ± 15.1 | 51.6 ± 17.6 | 0.1724 |
| Sex (male:female) | | 81:187 | 10:20 | 0.7263 |
| Clinical diagnosis | Graves’ disease/hyperthyroidism | 156 | 18 | 0.0956 |
|  | Painless/subacute thyroiditis | 88 | 8 |  |
|  | SNG^b^/MNG^c^ | 11 | 4 |  |
|  | Others | 13 | 0 |  |

a: standard deviation

b: single nodular goiter

c: multi-nodular goiter

Supplementary Table 2. Detailed demographics of thyroid SPECT/CT cases with partial CT for automatic thyroid segmentation and internal verification (n = 352)

|  | | For training  (n = 280) | For validation  (n = 36) | For internal verification  (n = 36) | *P*-value |
| --- | --- | --- | --- | --- | --- |
| Age [years] (mean±std^a^) | | 48.0 ± 15.6 | 46.0 ± 16.5 | 44.0 ± 14.9 | 0.2488 |
| Sex (male:female) | | 77:203 | 11:25 | 11:25 | 0.8761 |
| Clinical diagnosis | Graves’ disease/hyperthyroidism | 162 | 18 | 18 | 0.8073 |
|  | Painless/subacute thyroiditis | 94 | 15 | 14 |  |
|  | SNG^b^/MNG^c^ | 13 | 1 | 3 |  |
|  | Others | 11 | 2 | 1 |  |

a: standard deviation

b: single nodular goiter

c: multi-nodular goiter

Supplementary Table 3. Detailed demographics of salivary SPECT/CT cases for external verification (n = 29)

|  | | For external verification (n = 29) |
| --- | --- | --- |
| Age [years] (mean±std^a^) | | 53.1 ± 13.7 |
| Sex (male:female) | | 11:18 |
| Clinical diagnosis | Xerostomia | 11 |
|  | Salivary gland tumor | 7 |
|  | Head and neck cancer | 9 |
|  | Others | 2 |

a: standard deviation

**Quantitative thyroid single-photon emission computed tomography/computed tomography (SPECT/CT) protocol**

The preparation required no diet control. First, Tc-99m pertechnetate (185 MBq) was eluted from the Mo-99/Tc-99m generator (Unitech Technetium-99m generator, Samyoung Unitech) and was intravenously injected into the patients. Twenty minutes later, an anterior planar image was obtained for 1 min with neck extension. Immediately after planar image acquisition, SPECT and CT were consecutively performed without neck extension using SPECT/CT scanners (NMCT670 or NMCT670pro, GE) equipped with low-energy high-resolution collimators. The SPECT acquisition conditions were as follows: primary energy window peak at 140 keV (20% window of 126 keV–154 keV), scatter window peak at 120 keV (10% window of 115 keV–125 keV), and continuous mode acquisition for 1 min without body contour option. The acquisition zoom factor was set to 1.5. The CT acquisition parameters were as follows: tube voltage 120 kVp, tube current 30 mA, helical mode acquisition with detector collimation (16 x 1.25 = 20 mm), helical thickness 2.5 mm, table speed 37 mm/sec, table feed per rotation 18.75 mm/rot, tube rotation time 0.5 sec, and pitch 0.938:1.

The radioactivity and its measurement time were recorded before and after the injection of Tc-99m pertechnetate to quantify %thyroid uptake. We used a dose calibrator (CRC-15R, CAPINTEC) that had been daily calibrated using the National Institute of Standards and Technology traceable Co-57 source to measure radioactivity. Human experts who had been working on thyroid segmentation for 2 years (JHK and JHY) carefully segmented the thyroid on the CT images of the thyroid SPECT/CT upon dedicated quantitative software (Q.Metrix, GE), generating segmentation maps of the thyroid. System sensitivity of SPECT/CT scanners for Tc-99m was 152.5 cpm/μCi (from October 2015 to August 2017) and subsequently 151.8 cpm/μCi (since September 2017) for NMCT670, and 152.8 cpm/μCi (from December 2016 to August 2019) and then 149.3 cpm/μCi (since September 2019) for NMCT670pro.

**Quantitative salivary gland SPECT/CT protocol**

Patients fasted for at least 2 hours before SPECT/CT. Subsequently, Tc-99m pertechnetate (555 MBq), eluted from the Mo-99/Tc-99m generator (Unitech Technetium-99m generator, Samyoung Unitech), was intravenously injected. SPECT/CT was performed 20 min later. The same SPECT/CT scanners (NMCT670 or NMCT670pro, GE) equipped with LEHR collimators were used to perform a 1-min continuous mode SPECT without the body contour option. The primary peak and scatter peak energies were 140 keV with a 20% window (126 keV–154 keV) and 120 keV with a 10% window (115 keV–125 keV), respectively. The acquisition zoom factor was set to 1.5. CT acquisition parameters were tube voltage 120 KVp, tube current 30 mA, detector collimation 16 × 1.25 = 20 mm, helical thickness 2.5 mm, table speed 37 mm/sec, table feed per rotation 18.75 mm/rot, tube rotation time 0.5 sec, and pitch 0.938:1. SPECT and CT acquisition/reconstruction conditions were exactly the same as thyroid SPECT/CT.

**Loss functions**

The L_1_ and L_2_ loss functions were defined as follows:

$$L_{1}=\sum\left| G\left( X \right)-Y \right|$$

$$L_{2}=\sum{(G\left( X \right)-Y)}^{2}$$

The gradient difference loss (GDL) term used to compensate for imaging blurring by L_2_ loss effects is defined as follows:

$$L_{GDL}=\sum{(\left| \nabla G(X) \right|-\left| \nabla Y \right|)}^{2}$$

where ∇ is the image gradient operator.

**Evaluation of outcomes**

*R^2^*, mean square error (MSE), and %normalized mean absolute error (%NMAE) were defined as follows:

$$R^{2}=1-\frac{\sum{((G\left( X \right)-Y)}^{2}}{\sum{(Y-\bar{Y})}^{2}}$$

$$MSE=\frac{1}{No. of Voxels}\sum{((G\left( X \right)-Y)}^{2}$$

$$\%NMAE=\frac{1}{No. of Voxels}\sum\frac{\left| G\left( X \right)-Y \right|}{\max\left( Y \right)-min(Y)}$$

where Y is the target (i.e.). original μ-map), $\bar{Y}$ is the mean of Y, and G(X) is the synthetic μ-map from the SPECT input X.

Dice similarity coefficient (DSC) was defined as follows:

$$DSC((G\left( X \right),Y)=\frac{2\times\left| G\left( X \right)\cap Y \right|}{\left| G(X) \right|+\left| Y \right|}$$

where G(X)∩Y is the element-wise product of G(X) and Y.

95% Hausdorff distance was defined as follows:

$$d_{H95}\left( X,Y \right)=max(d_{XY},d_{YX})$$

where d_H95_ is the 95^th^ percentile of the maximum distance between the X (manual segmentation map) and Y (automatic segmentation map).
